# Lipidomic Analysis of Plastidial Octanoyltransferase Mutants of *Arabidopsis thaliana*

**DOI:** 10.3390/metabo9100209

**Published:** 2019-09-29

**Authors:** Raquel Martins-Noguerol, Antonio Javier Moreno-Pérez, Sebastien Acket, Salim Makni, Rafael Garcés, Adrián Troncoso-Ponce, Joaquín J. Salas, Brigitte Thomasset, Enrique Martínez-Force

**Affiliations:** 1 Instituto de la Grasa-CSIC, Building 46, UPO Campus, Ctra. de Utrera km 1, 41013 Seville, Spain; rmnoguerol@ig.csic.es (R.M.-N.); ajmoreno@ig.csic.es (A.J.M.-P.); rgarces@ig.csic.es (R.G.); jjsalas@ig.csic.es (J.J.S.); 2 Alliance Sorbonne Université, Université de Technologie de Compiègne, Génie Enzymatique et Cellulaire (GEC), UMR-CNRS 7025, CS 60319, 60203 Compiègne CEDEX, France; sebastien.acket@utc.fr (S.A.); salim.makni@utc.fr (S.M.); adrian.troncoso-ponce@utc.fr (A.T.-P.); brigitte.thomasset@utc.fr (B.T.)

**Keywords:** octanoyltransferases, protein lipoylation, triacylglycerol species, polar lipids, fatty acid biosynthesis

## Abstract

Plant de novo fatty acid synthesis takes place in the plastid using acetyl-coenzyme A (acetyl-CoA) as the main precursor. This first intermediate is produced from pyruvate through the action of the plastidial pyruvate dehydrogenase complex (PDH), which catalyses the oxidative decarboxylation of pyruvate to produce acetyl-CoA, CO_2_, and NADH. For the proper functioning of this complex, lipoic acid is required to be bound to the dihydrolipoamide S-acetyltransferase E2 subunit of PDH. Octanoyltransferase (LIP2; EC 2.3.1.181) and lipoyl synthase (LIP1; EC 2.8.1.8) are the enzymes involved in the biosynthesis of this essential cofactor. In *Arabidopsis* plastids, an essential lipoyl synthase (*At*LIP1p) and two redundant octanoyltransferases (*At*LIP2p1 and *At*LIP2p2) have been described. In the present study, the lipidomic characterization of *Arabidopsis* octanoyltransferase mutants reveals new insight into the lipoylation functions within plastid metabolism. Lipids and fatty acids from mature seeds and seedlings from *Atlip2p1* and *Atlip2p2* mutants were analysed by gas chromatography (GC) and liquid chromatography–electrospray ionization high-resolution mass spectrometry (LC-ESI-HRMS2), the analysis revealed changes in fatty acid profiles that showed similar patterns in both mutant seeds and seedlings and in the lipid species containing those fatty acids. Although both mutants showed similar tendencies, the lack of the *At*LIP2p2 isoform produced a more acute variation in its lipids profile. These changes in fatty acid composition and the increase in their content per seed point to the interference of octanoyltransferases in the fatty acid synthesis flux in *Arabidopsis thaliana* seeds.

## 1. Introduction

In plants, the main sites of fatty acid synthesis are plastids [1], where acetyl-coenzyme A (acetyl-CoA) is the first intermediate [2,3,4]. This acetyl-CoA is generated by the activity of the pyruvate dehydrogenase (PDH) complex. This enzymatic complex is regulated by lipoylation of one of its subunits, dihydrolipoamide dehydrogenase (E2 subunit). Therefore, E2 needs to be lipoylated for correct activity of the full complex.

Lipoic acid biosynthesis is performed by the activities of octanoyltransferase (LIP2) and lipoyl synthase (LIP1) enzymes. The lipoylation machinery is not fully elucidated but evidences suggest a two-step lipoylation pathway through the joint activity of both enzymes: First LIP2 proceeds transferring the octanoyl moiety from octanoyl-acyl carrier protein (octanoyl-ACP) to the ε-amino group of a highly conserved residue of Lys, close the N-terminus of E2-PDH, via amide link [5]. Then, LIP1 inserts two sulphur atoms from S-adenosyl methionine (SAM) into C6 and C8 of the octanoyl chain forming the 1,2-dithiolane ring of lipoic acid (LA) [6]. Unlike most cofactors, lipoic acid assembly on its cognate proteins constitutes an extraordinary/uncommon pathway.

This lipoylation machinery is responsible for the functionality of other complexes and enzymes involved in oxidative and carbon metabolism in most prokaryotic and eukaryotic organisms including: α-ketoglutarate dehydrogenase (KGDH), branched-chain α-ketoacid dehydrogenase (BCDH), acetoin dehydrogenase (AoDH), and the glycine cleavage complex (glycine decarboxylase, GDC) [7,8,9,10]. Substantial knowledge about lipoylation processes has been obtained from studies with bacteria [11] but little is known in higher eukaryotes.

In plants, PDH, KGDH, and GDC complexes are located within the mitochondria, but PDH also occurs in plastids. In plastids, where no evidence exists for lipoic acid transport, E2-PDH is thought to be lipoylated through the activities of specific plastidial isoforms of LIP1 and LIP2. One lipoyl synthase and two octanoyltransferases have been found in plastids from *Arabidopsis thaliana* (AtLIP1p, AtLIP2p1, and AtLIP2p2) [12]. AtLIP1p has been demonstrated to be essential for embryo development, meanwhile both octanoyltransferases seems to be redundant, the homozygous disruption of the *Lip2p1* gene had no visible effects on growth and development of *Arabidopsis* plants if LIP2p2 is present [12]. Plants are not able to survive without functional plastidial E2-lipoylated PDH [13], highlighting the crucial role played by this pathway upon fatty acid synthesis and regulation.

Notwithstanding the importance of this metabolic pathway within plastids, to our knowledge this is the first study shedding light upon the influence of octanoyltransferase enzymes on plant lipid metabolism.

## 2. Results

### 2.1. Total Fatty Acid Composition of Mutant Seeds

**Figure 1:**
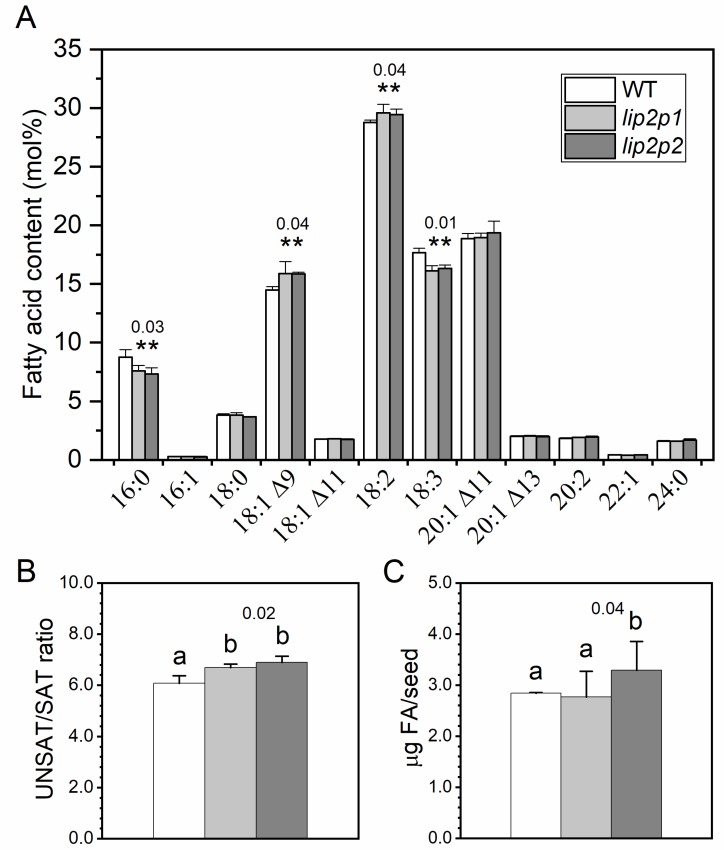
Fatty acids composition (**A**), unsaturated/saturated fatty acid ratio (**B**) and fatty acid content per seed (**C**) of mature seeds from control (wild type, WT) and octanoyltransferase mutants (*lip2p1* and *lip2p2*) lines. The data correspond to the mean ± SD of three independent measurements. ** At the 0.05 level, data are significantly different (showing *p* values). Different letters reflect the mean statistical difference (*p* < 0.05). 16:0, palmitic acid; 16:1, palmitoleic acid; 18:0, stearic acid; 18:1^Δ9^, oleic acid; 18:1^Δ11^, asclepic acid; 18:2, linoleic acid; 18:3, linolenic acid; 20:1^Δ11^, gondoic acid; 20:1^Δ13^, eicosenoic acid; 20:2, eicosadienoic acid; 22:1, erucic acid; 24:0, lignoceric acid.

Fatty acid profiles of mutant seeds harvested from confirmed mutant homozygous plants were analysed (Figure 1A). Variations in some fatty acid species were detected; a slight but significant decrease of 16:0 and 18:3 compensated with an increase of 18:1^Δ9^ and 18:2 (*p* < 0.05). Consequently, the observed unsaturated/saturated fatty acid ratio was increased in both mutant seeds in comparison with the wild type (Figure 1B). This increase was minor but significant (*p* < 0.05). In addition, significant differences were detected in total micrograms of fatty acids/seed between *lip2p2* mutant and wild-type seeds (Figure 1C). No differences were detected in mutant seed external phenotypes in comparison with wild type, with the weight of the mutant seeds being quite similar to the one for Col-0 (*lip2p1* 17.33 ± 2.31 μg, *lip2p2* 18.67 ± 2.31 μg, and wild type 17.3 ± 1.15 μg).

### 2.2. Lipid Analysis of Mutant Seedlings

**Figure 2:**
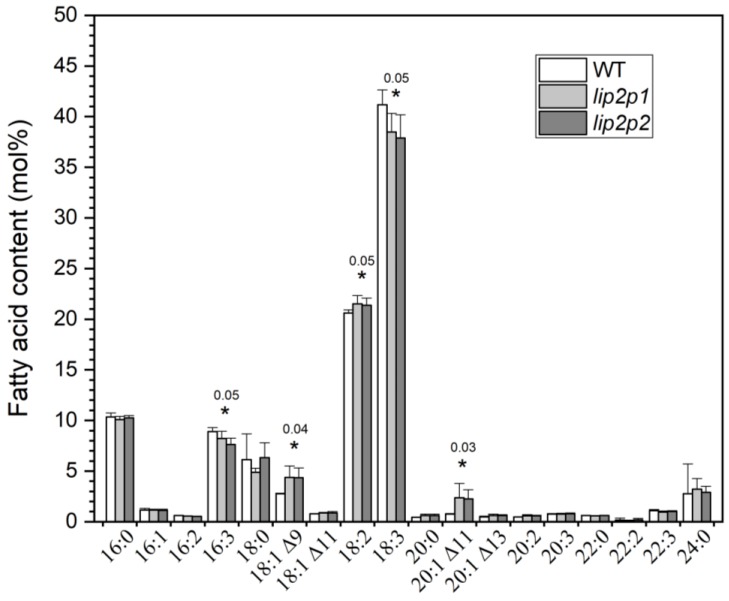
Fatty acid composition of seedlings from control (WT) and octanoyltransferase mutants (*lip2p1* and *lip2p2*) lines. The data correspond to the mean ± SD of three independent measurements. * At the 0.05 level, data are significantly different (showing *p* values). 16:0, palmitic acid; 16:1, palmitoleic acid; 16:2, palmitolinoleic acid; 16:3, palmitolinolenic acid; 18:0, stearic acid; 18:1^Δ9^, oleic acid; 18:1^Δ11^, asclepic acid; 18:2, linoleic acid; 18:3, linolenic acid; 20:0, arachidic acid; 20:1^Δ11^, gondoic acid; 20:1^Δ13^, eicosenoic acid; 20:2, eicosadienoic acid; 20:3, eicosatrienoic acid; 22:0, behenic acid; 22:2, docosadienoic acid; 22:3, docosatrienoic acid; 24:0, lignoceric acid.

Lipid species profiles and fatty acid compositions of mutant seedlings were also analysed. Although no differences were found in the lipid species, fatty acid profiles showed similar results to the ones found between wild-type and mutant seeds, with a decrease of 16:3 and 18:3 content and increase of 18:1, 18:2, and 20:1 (Figure 2). Again, no external phenotypic differences were detected in mutant seedlings in comparison with wild type.

### 2.3. Analysis of Lipid Components in Mutant Seeds

**Figure 3:**
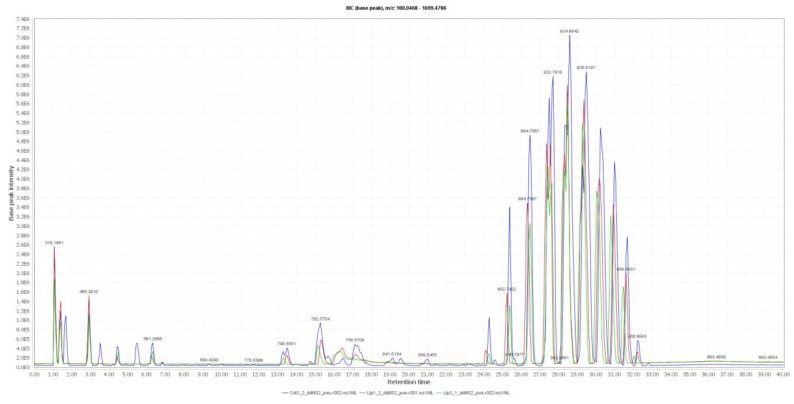
Example of total ion current map of lipids from control and mutant lines of *Arabidopsis* mature seeds in positive-ion mode.

Samples in the control line Col-0 and mutant lines were detected by liquid chromatography coupled to high-resolution mass spectrometry (LC-ESI-HRMS2). The samples were randomized in both negative- and positive-ion mode. The raw data were analysed using MZmine v2.39 [14] and features annotated using databases of over 250,000 MS/MS spectra, lipid match [15], and lipid blast [16]. An example of the total ion current map (TIC) in positive mode is shown in Figure 3. One hundred and thirty seed lipid molecules were covered in both ion modes (data not shown): triglyceride (TG); diglyceride (DG); lysophosphatidylcholine (lysoPC); phosphatidylcholine (PC), and phosphatidylethanolamine (PE).

### 2.4. Non-Targeted Lipidomic Metabolic Analysis

**Figure 4:**
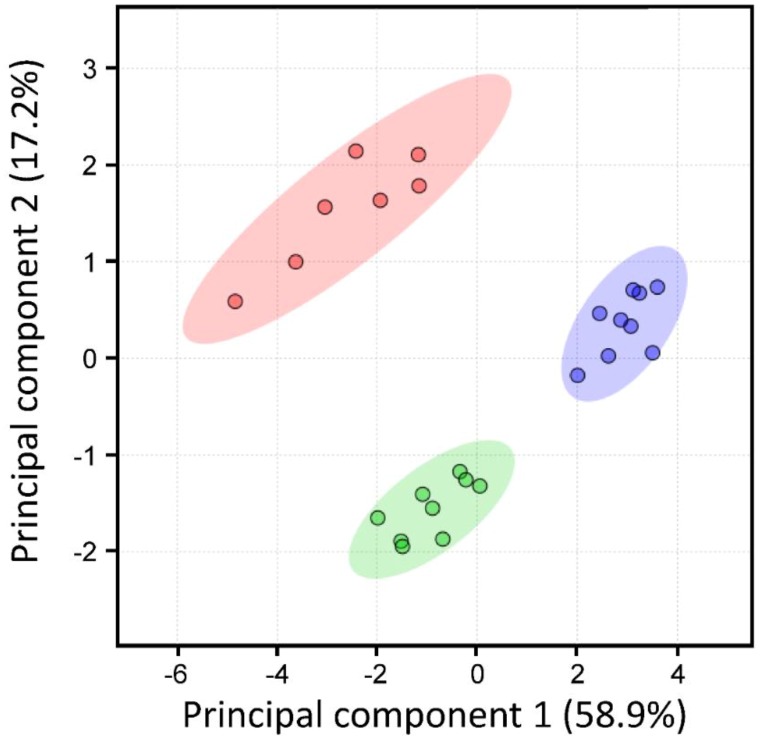
Principal component analysis (PCA) plot showing the similarity relations among *Arabidopsis* mature seeds from control and octanoyltransferase mutants. These two components explain 76.1% of the variance. Samples from control line are shown as red circles; those from *lip2p1* or *lip2p2* mutants as green and purple circles, respectively.

The lipid metabolites between both *lip2p* insertional mutants and control *Arabidopsis* line were compared. All the resulting data in both ion modes were subjected to principal component analysis (PCA) to determine the whole experimental variation (Figure 4). Each dot in the figure represents a sample. PCA model of global lipid changes exposed consistent separation of mutant groups from Col-0 controls suggesting that the different genotypes have metabolic differences.

### 2.5. Analysis of Differential Lipid Metabolites

**Figure 5:**
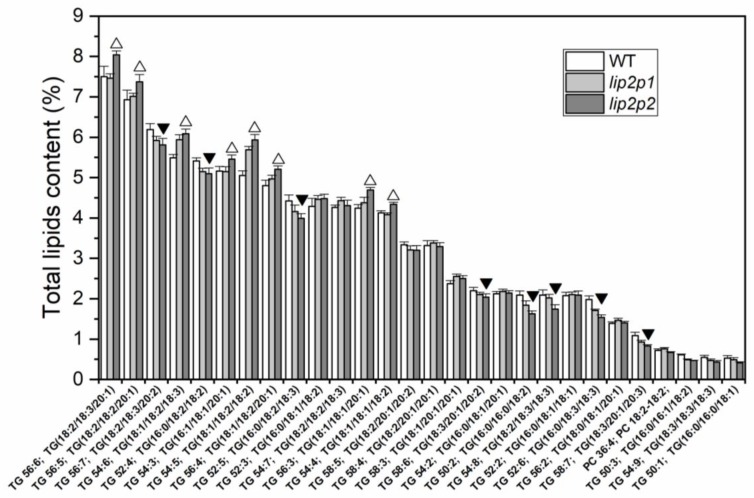
Representative TAG and glycerolipid species of the differences found between *Arabidopsis* control (WT, white columns) and octanoyltransferase mutants (*lip2p1*, grey, and *lip2p2*, dark grey) mature seeds. Data are average values ± SD of three biological replicates. △▼ At the 0.05 level, data are significantly different for *lip2p2* values.

**Figure 6:**
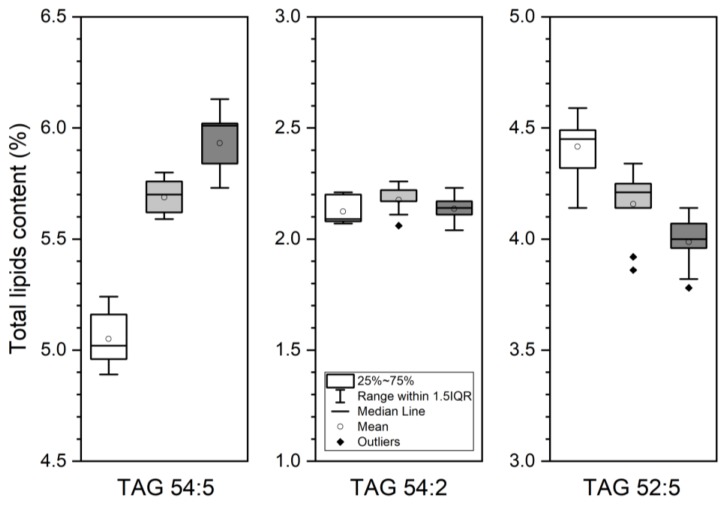
Representative changes observed in TAG 54:5 (18:1/18:2/18:2), 54:2 (16:0/18:1/20:1), and 52:5 (16:0/18:2/18:3) when comparing *Arabidopsis* control (WT, white) and octanoyltransferase mutants (*lip2p1*, grey, and *lip2p2*, dark grey) mature seeds. Data are average values ± SD of three biological replicates with two technical ones.

**Figure 7:**
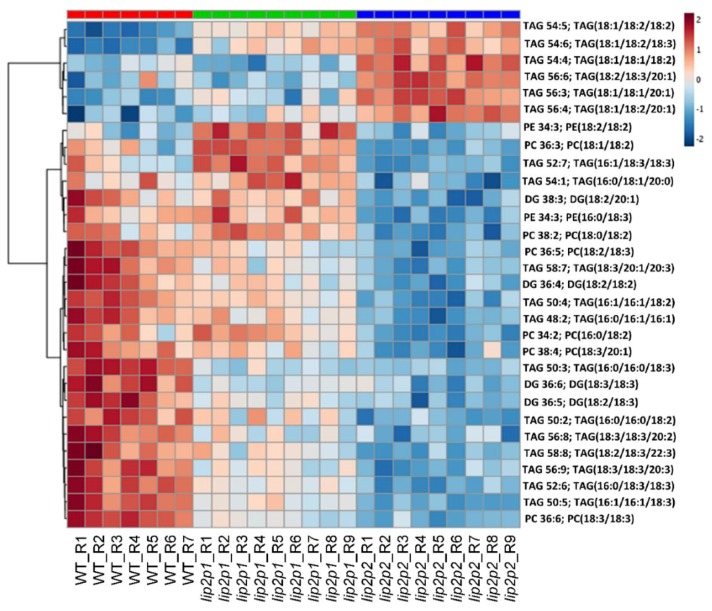
Heatmap of the most statistically different lipids species (right) comparing the control (WT, red) and the octanoyltransferase mutant mature seeds (*lip2p1*, green, and *lip2p2*, blue). TOP 25 *p* < 0.001 ANOVA Tukey non-parametric.

Lipidomic analysis of control and mutant *Arabidopsis* seeds was performed. A total of 67 lipids were annotated. Figure 5 shows the 28 most abundant lipids species in the three lines, pointing those decreasing or increasing in mutant seeds in comparison with wild-type (WT) data. The results show that there is a significant difference between control and mutant groups (*p* < 0.05). Figure 6 represents a box–whisker plot of normalized peak intensity of three representative triacylglycerol (TAG) species which increased, decreased, or maintained their content. The differential lipids were clustered and the resulting heatmap is shown in Figure 7. Each square in the heatmap represents the corresponding intensity value of a lipid in each group (red and blue represent an increase or a decrease in concentration respectively). Compared with the control group some lipids in the mutants show an upward trend and others a downward trend, but the results evidence a more pronounced effect in the case of *lip2p2* mutant seeds.

## 3. Discussion

In plants, as in other eukaryotes, most lipoate-dependent enzymes are located in the mitochondrion. The PDH complex is an exception because it also occurs in plastids [17], which are the major site for de novo fatty acid biosynthesis in plants. This enzymatic complex is essential for the supply of acetyl-CoA to the above-mentioned biosynthetic pathway [18]. It contains a lipoate-containing the dihydrolipoamide S-acetyltransferase (E2) subunit, which needs to be lipoylated for the correct function of PDH. Because plants are not able to survive without functional E2-lipoylated plastidial PDH [13], plastidial PDH-E2 lipoylation is a vital process.

In plastids of *Arabidopsis* a two-step mechanism for lipoylation has been proposed, which involves a lipoyl synthase (LIP1p) [19] and two octanoyltransferase enzymes (LIP2p1 and LIP2p2) [12,20]. LIP1p disruption is embryo lethal [12]. However, the homozygous disruption of the *LIP2p1* gene shows no phenotypic effects on growth and development, as in the case of *LIP2p2* disruption, although the combined inactivation of both genes resulted in embryonic lethality. This fact demonstrates that both octanoyltransferases are redundant in *Arabidopsis* plants [12]. Nevertheless, to the best of our knowledge, no studies exist about the involvement of these mutations in the context of lipid biosynthesis.

In the present work, the lipidomic characterizations of these two different mutants, *lip2p1* and *lip2p2*, were performed, the data obtained provide a basic groundwork for the understanding of this essential lipoylation pathway and for further biotechnological approaches aimed to modify lipid biosynthesis and accumulation in plants.

Based on the previously described redundancy, the seed oil composition from *Arabidopsis* mutants impaired in one of the two octanoyltransferase isoforms is expected to be similar to WT plants, although a tendency for seeds with higher fatty acid content in *lip2p2* mutants was observed (Figure 1C). However, unexpected differences were obtained when total fatty acid profiles were analysed, both mutant seeds, *lip2p1* and *lip2p2*, showed a significant decrease (*p* < 0.05) in 16:0 and 18:3 fatty acids accompanied by an increase in 18:1 and 18:2 content (Figure 1A). Consequently, significant changes were observed in the unsaturated/saturated fatty acid ratio, which increased in both *lip2p1* and *lip2p2* mutants in comparison with wild-type seeds (Figure 1B). On the other hand, the total amount of fatty acids per seed was higher in *lip2p2* than in *lip2p1* and WT seeds (Figure 1C).

When fatty acids were determined in seedlings from the three lines (WT, *lip2p1*, and *lip2p2* lines) similar results to the ones found in mature seeds were observed: Reduction of 18:3 and increase in 18:1 and 18:2 content. In this case, no differences were observed with respect to palmitic acid (16:0) but differences in 16:3 levels were found. In mature seeds, the main lipids are triacylglycerols, in contrast to seedlings where polar lipids, synthetized inside the chloroplast, are the most abundant lipids. For this reason, most of the palmitic acid is not exported to the cytoplasm via acyl-ACP thioesterases but is desaturated up to 16:3 in the chloroplast. In seedlings, a reduction in 16:0 will be observed as a reduction in 16:3, its final product. In addition, an increase of 20:1 was observed as consequence of the 18:1 increment.

Accordingly, confirming results obtained by GC analysis of total fatty acids, lipidomic analysis of these lines showed changes in those lipid species containing fatty acids with variations in their content (Figure 5). Consequently, between the triacylglycerol species increasing content in mutant lines were found: 18:2/18:3/20:1, 18:2/18:2/20:1, 18:1/18:2/18:3, 16:1/18:1/20:1, or 18:1/18:2/18:2 (Figure 6, TAG 54:5). On the other hand: 18:2/18:3/20:2, 16:0/18:2/18:3 (Figure 6, TAG 52:5), 16:0/16:0/18:2, 18:2/18:3/18:3, or 16:0/18:3/18:3 decreased their content. When principal component analysis (PCA) was carried out on lipidomic data, three clear groups defining each of the genotypes (control, *lip2p1*, and *lip2p2*) were obtained (Figure 4). Furthermore, the heatmap of the statistically different lipid species showed clear differences between the genotypes, although also points to the fact that *lip2p2* shows more pronounced differences with respect to control values (Figure 7).

To explain the changes induced in the plant lipids by the silencing of Lip2p enzymes we must consider that the substrate of octanoyltransferases is octanoyl-ACP, an intermediate for the synthesis of fatty acids shorter than palmitic, stearic, and oleic acids, the main fatty acids produced during de novo intraplastidial fatty acid biosynthesis. This implies direct competition for this substrate with members of the fatty acid synthase (FAS) complex. More precisely, octanoyl-ACP is the product of the enoyl reductase activity and a substrate for β-ketoacyl-ACP synthase I (KASI) which will initiate an additional round of elongation. In other words, its activity interferes with the de novo synthesis of fatty acids, but it is also necessary for this process because pyruvate dehydrogenase (PDH) depends on lipoic acid synthesis. Furthermore, given the mechanism of the octanoyltransferase activity, a two-step catalytic mechanism by means of a thioester acyl intermediate [21], it can be speculated that a pool of octanoyl-charged octanoyltransferases could exist, ensuring the availability of octanoate ready for lipoic acid synthesis, avoiding any shortage of the metabolite for the synthesis of the essential PDH complex.

**Figure 8:**
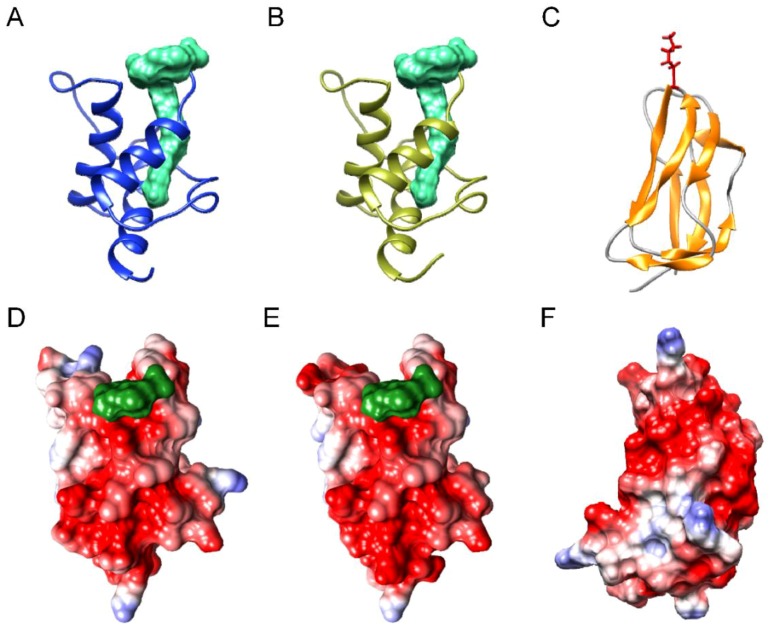
Proposed structural models for *Arabidopsis* acyl-carrier protein ACP1 (**A**,**D**), ACP2 (**B**,**E**), and plastid E2 subunit of pyruvate dehydrogenase LTA2 (**C**,**F**), based on those of *Spinacia oleracea* acyl-ACP (Protein Data Bank accession 2fva) [22] and human pyruvate dehydrogenase complex (Protein Data Bank accession 1y8n) [23]. Panels (**A**–**C**) are ribbon diagrams in which the acyl chains are shown in green (**A**,**B**) and lysine residue in red (**C**). Panels (**D**–**F**) are views of the molecular surfaces showing the electrostatic potentials according to Coulomb’s laws with extensive negatively charged patches near the acyl chain insertion (in acyl-ACPs, panels **D**,**E**) or the lysine residue (in LTA2).

**Figure 9:**
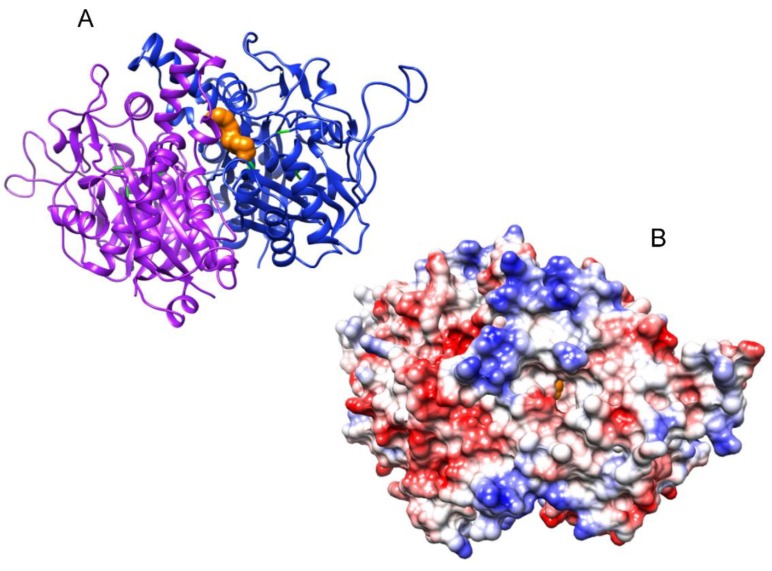
Proposed structural models for *Arabidopsis* β-ketoacyl-ACP synthase I (*At*KASI; panels **A**,**B**) homodimer based on that of *Escherichia coli* FabF (2gfy) [25]. Panel (**A**) is a ribbon diagram in which the catalytic triad residues are in green and the acyl chain in orange. Panel (**B**) shows the molecular surface showing the electrostatic potentials according to Coulomb’s laws with a positively charged patch pointing to the entrance of the substrate binding pocket (showing the acyl chain in orange).

**Figure 10:**
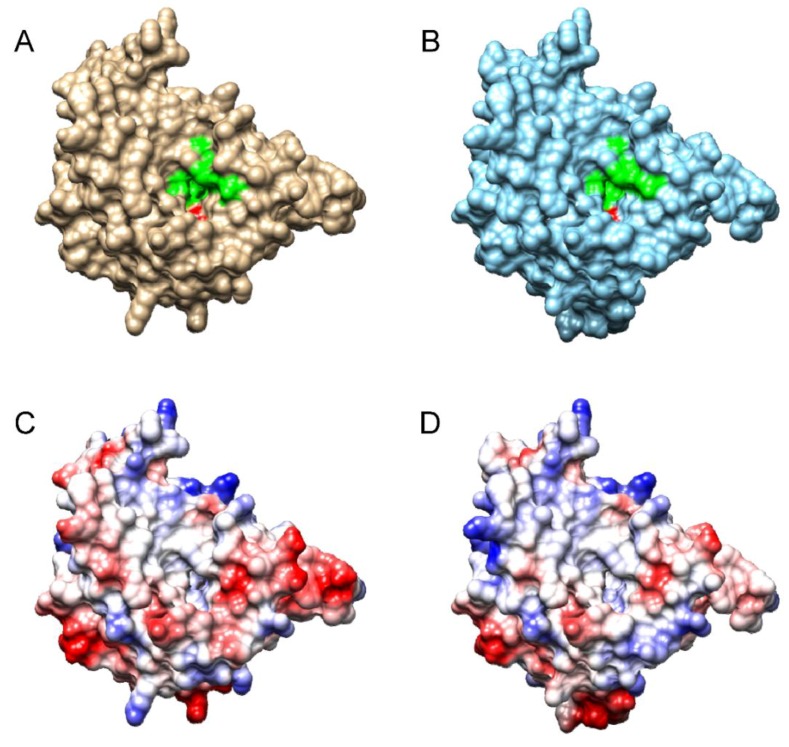
Proposed structural models for *Arabidopsis* LIP2p1 (**A**,**C**) and LIP2p2 (**B**,**D**) based on that of *Mycobacterium tuberculosis* octanoyl-ACP (acyl carrier protein) acyltransferase (1w66) [21]. Panels (**A**,**B**) are views of the molecular surfaces in which the residues involved in the active site are shown in green and the catalytic lysine residue in red. Panels (**C**,**D**), views of the molecular surfaces showing the electrostatic potentials according to Coulomb’s laws.

In addition to this initial substrate (octanoyl-ACP), octanoyltransferases must also recognize its target protein, more specifically a lysine residue in the N-terminal region of the PDH E2 subunit. For this reason, and, since the reaction takes place by means of the sequential recognition of both proteins (ACP and E2) these proteins must have features in common. As shown in Figure 8, the surface of both is negatively charged: around the acyl chain of the acyl-ACPs (Figure 8D,E) and around the lysine residue of the E2 subunit (Figure 8F).The importance of positively charged patches for enzyme-substrate recognition in the acyl-ACP thioesterases has been already described [24]. In fact, as Figure 9 shows, these positively charged patches around the active site of the enzyme are also found in β-ketoacyl-ACP synthase I. When examining the predicted structure (Figure S2) and surfaces for both *Arabidopsis* octanoyltransferases (Figure 10), we observed differences in charge around the active site, in particular, *lip2p2* presents a more positive/less negative charge than *lip2p1*, which would indicate that both, even presenting a 72.14% identity (Figure S2), will present different affinity for their substrates explaining in part the differences found between mutants in this study. Furthermore, although both genes are expressed in all organs they are found at different ratios: *LIP2p1* with higher expression in leaves and roots and *LIP2p2* in siliques and flowers [12].

Therefore, metabolic differences between the mutants lacking one of these enzymes are expected not only due to structural differences but also due to expression levels. The analysis of the results points to a greater effect on lipids species, although changes going in the same direction were observed, in the absence of LIP2p2 rather than when lacking LIP2p1 ( Figure 1, Figure 2, Figure 4, Figure 5 and Figure 7), so we will use this mutant more pronouncedly to explain possible causes of observed phenotypes (lipid differences). Firstly, the reduction of this transferase activity, depending on its total contribution, would entail a greater availability of octanoyl-ACP for the synthesis of fatty acids, leading to expected higher synthesis of these compounds, as observed in *lip2p2* seeds. In addition, competition with FAS complex enzymes, which could be being limited by this protein, would be reduced. The increase of the fatty acids per seed (Figure 1C) and the reduction of palmitic acid content (due to greater FAS activity, Figure 1A) would support the above statement. This would produce a greater synthesis of oleic as the end product of the intraplastidial fatty acid biosynthesis.

The expected lack of alterations in the levels of expression/activity of the endoplasmic reticulum enzymes, which carry out subsequent modifications of oleic acid or the assembly of triacylglycerols, due to minor changes in fatty acids coming out from the plastid, together with its greater synthesis in octanoyltransferase mutants, causes a higher incorporation of oleic and a relative reduction of linolenic acid in seed triacylglycerols (Figure 1A). Meanwhile, in seedlings, the consequence is an increment of oleic, linoleic, and gondoic acid and a reduction of linolenic acid (Figure 2). The opposite effect, to the one observed in octanoyltransferase mutants (increase of 18:3 and reduction of 18:1 and 18:2 and 20:1), occurs when there is a reduction of the synthesis of fatty acids in mutants with lower levels of KASI [26] and KASIII activities [27], confirming/ratifying that the activity of octanoyltransferase must produce a slowing of the route of fatty acid synthesis, which is partially relieved in the studied mutants. The changes in the profiles of these fatty acids produced in turn cause the observed changes in glycerolipids and glycerophospholipids species, reducing those species with 16:0 or 18:3 and increasing those with 18:1 or 18:2.

## 4. Materials and Methods 

### 4.1. Plants and Growth Conditions

Plants used in this study were *A. thaliana* ecotype Columbia (Col-0) and two SALK T-DNA insertion lines obtained from the Nottingham Arabidopsis Stock Centre: SALK_ 085456C with disrupted *LIP2p1* gene (AT4G31050) and SALK_038140C with disrupted *LIP2p2* (AT1G47578). Plants were grown in chambers under controlled conditions with a photoperiod of 16/8 h light/dark (22/20 °C) and 250 µmol m^−2^ s^−1^.

### 4.2. Confirmation of Homozygous Lines Lip2p1 and Lip2p2

In both *lip2p* insertional mutants, T-DNA insertions were confirmed in genomic DNA by PCR using a specific primer for the SALK T-DNA left border (LBb1.3 for SALK lines; all primers used in this study are listed in Table S1) and specific primers for *LIP2p1* (AtLIP2p1-RP) and *LIP2p2* (AtLIP2p2-RP). Once T-DNA insertion was confirmed, absence of intact *LIP2p1* or *LIP2p2* genes was also confirmed by PCR with specific primers AtLIP2p1-LP/AtLIP2p1-RP and AtLIP2p2-LP/AtLIP2p2-RP, respectively. In addition, absence of transcripts of *LIP2p1* and *LIP2p2* in each mutant was confirmed by RNA extraction and cDNA synthesis. RT-PCR reactions with specific primers were performed to confirm the absence of *LIP2p1* (primers AtLIP2p1-transcript-F/AtLIP2p1-transcript-R) and *LIP2p2* (AtLIP2p2-transcript-F/AtLIP2p2-transcript-R). Genomic DNA and cDNA from wild-type plants were used in each case as control in PCR reactions. Genotyping data are shown in Figure S1.

### 4.3. Total Fatty Acid Composition of Mutant Seeds

Three replicates of 50 mature seeds from confirmed homozygous mutants (*lip2p1* or *lip2p2*), and from wild-type plants were used for total fatty acid analysis. Briefly, 2 mL methylation mixture containing methanol/toluene/sulphuric acid (88:10:2, *v*/*v*/*v*) and 60 μg of heptadecanoic acid as internal standard was added to each sample and mixture was incubated at 80 °C for 1 h for lipid methylation. Then, 2 mL hexane was added for fatty acid extraction, the upper phase was recovered and then washed with 1 mL Na_2_SO_4_ 6.7%. The upper phase was recovered, and the solvent was evaporated under nitrogen. The fatty acid methyl esters were resuspended in 200 µL heptane for gas chromatography. The statistical analysis was performed with IBM SPSS Statistics software.

### 4.4. Lipid Analysis of Mutant Seedlings

One-week-old *Arabidopsis* seedlings growing on Petri plates were used for lipid extraction. Three replicates of approximately 230 mg were performed. Seedlings were introduced in tubes with 2 mL isopropanol and 111 μg of heptadecanoic acid (internal standard) and then heated at 80 °C for 15 min. Accordingly, seedlings were homogenized with sand and 3 mL hexane was added for lipid extraction. The upper phase was recovered and then washed with 2.5 mL Na_2_SO_4_ 6.7%. The upper phase was recovered again. Re-extraction of aqueous phase was performed with 3.7 mL hexane:isopropanol (7:2). Both upper phases were combined and then evaporated under nitrogen. Total lipids were resuspended in 20 µL heptane. Half of the lipids was used for gas chromatography and the other half was used for methylation and analysis of fatty acids of methyl esters. For methylation, the protocol was the same described above for total fatty acid composition of mutant seeds.

### 4.5. Sample Preparation for Lipidomics and LC-HRMS2 Analyses

Lipids were extracted as described in Folch et al. [28] with modifications. Lipids from 20 mg of dry *Arabidopsis* seed were extracted using 1 mL of chloroform:methanol (2:1) with 1 mM butylhydroxytoluene, BHT, and 400 µL H_2_O. The mixture was incubated for 2 h in ice, and then the samples were centrifuged at 13,000 rpm for 5 min at 4 °C. The lower phase (organic layer) was recovered and the lipid extraction was repeated. Both lower phases were combined, dried under nitrogen, and then resuspended in 200 µL isopropanol and diluted by four times.

LC was performed using an HPLC 1290 (Agilent Technologies). For lipid separation, a C18 Hypersil Gold (100 × 2.1 mm, 1.9 μm, Thermo Fisher) was used at 50 °C with an elution gradient of a solution of 10 mM ammonium formate and 0.1% formic acid (ACN: H_2_O = 60:40, *v*/*v*; solvent A) and a solution of 10 mM ammonium formate and 0.1% formic acid (IPA:ACN:H_2_0 = 90:8:2, v/v; solvent B). The gradient conditions were: A = 0–2 min 68%, 2–8 min 60%, 8–10 min 55%, 10–16 min 50%, 16–22 min 40%, 22–28 min 30%, 28–35 min 20%, 35–40 min 0%. Finally, B was returned to 40% over the next 0.1 min and equilibrated for 10 min for the next injection. The flow rate was set at 0.25 mL min^−1^ and the injection volume was 2 µL.

LC-ESI-HRMS2 analyses were performed by coupling the LC system to a hybrid quadrupole time-of-flight ultra-high definition (QToF) mass spectrometer Agilent 6538 (Agilent Technologies) equipped with an electrospray (ESI) dual source. The source temperature was 350 °C, fragmentor 150 V and the skimmer 65 V. The acquisition was made in full scan mode between 100 *m*/*z* and 1700 *m*/*z*, with a scan of two spectra per second. Selected parent ions were fragmented with collision energy fixed at 35 eV. MS2 scans were done of the sixth most intense ions above. Two internal reference masses were used for in-run calibration of the mass spectrometer (121.0509, 922.0098 in positive ion mode) and 112.9856, 1033.9881 in negative ion mode. The total LC-HRMS2 run was 40 min with 10 min of equilibration. MassHunter B.07 software allowed to control the parameters of the machine and acquired the data. Agilent-generated files corresponding to samples (*.d) were converted to *.mzXML files using MSConvert software [29]. Such converted files were processed using MZmine2 v2.39 [14]. The mass spectra were acquired using a dual electrospray ionization in positive- and negative-ion mode.

### 4.6. Data Processing and Annotation

The mass detection was realized keeping the noise level at 2.0E3 for MS1 and 0E00 for MS2 in centroid. The chromatogram builder was used using a minimum time span of 0.10 (min), a minimum height of 1.0E3, and *m*/*z* tolerance of 5 ppm. The chromatogram deconvolution was applied using the local-minimum search algorithm. The chromatographic threshold was 30.0%, the search minimum in RT range was 0.05 min with a minimum relative height of 5% and a minimum ratio of peak top/edge of 2. Peak duration range was 0.05–3 min. MS2 scans were paired using a *m*/*z* tolerance range of 0.05 Da and RT tolerance range of 0.1 min. Isotopologues were grouped using the isotopic peak grouper algorithm with a *m*/*z* tolerance of 0.008 and a RT tolerance of 0.3 min. A peak alignment step was performed using the join aligner module (*m*/*z* tolerance = 0.008, weight for *m*/*z* = 50, weight for RT = 50, absolute RT tolerance 2 min). Peak finder module was used with Intensity tolerance of 10%, *m*/*z* tolerance of 0.008, and retention time tolerance of 1.0 min. The resulting peak list was then filtered using the Peak list rows filter module with a Minimum peak in a row of 2, a Minimum peak in an isotope pattern of 2, and by keeping only peaks with MS2 scan (GNPS). The peak list was then exported to *.csv using the module “Export to CSV file”. Moreover, a *mgf was exported using the module: ”Export for/Submit to GNPS”. The peak list was annotated using a combination of two databases, lipid match [15], and lipid blast [16].

### 4.7. Statistical Analysis for Lipidomic Data

The statistical analysis for lipidomics was performed by MetaboAnalyst v3.0 [30]. For determination of statistical significance, the annotated features were first normalized using the sum of all peaks, then a log2 function, mean-centred and divided by the square root of the standard deviation of each variable. A PCA was performed to detect outliers. The heatmap was performed to detect the top 25 significant differences (<0.001) between the different *Arabidopsis* seed samples.

### 4.8. Protein Modelling

Homology modelling of the putative *At*LIP2p1 and *At*LIP2p2 protein structures was carried out with the DeepView and Swiss Model Workspace software [31] (http://www.expasy.org/spdbv/), using their protein sequences and the octanoyl-ACP acyltransferase crystal structure from *Mycobacterium tuberculosis* as a template (Protein Data Bank accession 1w66) [21]. The *At*LIP2p1 (K44-E268) and *At*LIP2p2 (K46-E271) residues were modelled against this template (Figure 10), with sequence identities of 38.05% and 34.95%, respectively. Critical residue mapping and visualization was performed using the UCSF Chimera package [32].

In a similar way, homology modelling of the putative *At*ACP1 and *At*ACP2 protein structures was carried out, using their protein sequences and the acyl carrier protein (ACP) crystal structure from *Spinacia oleracea* as a template (Protein Data Bank accession 2fva) [22]. The *At*ACP1 (A56-K136) and *At*ACP2 (A54-A135) residues were modelled against this template (Figure 8), with sequence identities of 60.49% and 58.54%, respectively. Furthermore, homology modelling of the N-terminal domain of the putative *At*LTA2 protein structure was carried out, using its protein sequence and the lipoyl domain 2 of human pyruvate dehydrogenase complex as a template (Protein Data Bank accession 1y8n) [23]. The *At*LTA2 (K54-A134) residues were modelled against this template (Figure 8), with sequence identities of 40.74%. Moreover, homology modelling of the putative *At*KASI protein structure was carried out, using its protein sequence and the 3-oxoacyl-ACP synthase (FabF) crystal structure from *Escherichia coli* as a template (Protein Data Bank accession 2gfy) [28]. The *At*KASI (K60-F487) residues were modelled against this template (Figure 9), with sequence identities of 60.49%.

## 5. Conclusions

The suppression of the expression of individual forms of plastidial lipoyl transferases in *A. thaliana* has an impact on the lipid composition of the plant seeds and seedlings. These changes involved a decrease in palmitic acid, 16:0, and polyunsaturated fatty acids at the expenses of oleate and significant increases of the seed oil content in the case of the form *At*LIP2p2. Lipidomic studies on the mutants confirmed those differences, showing the mutants’ higher contents of glycerolipid species containing oleate moieties. Furthermore, a PCA of seed lipids clustered the three lines studies in independent groups, clearly differentiated. All these results seemed to point that lipoate metabolism interferes with de novo fatty acid synthesis in *Arabidopsis* developing seeds and seedlings.

## Supplementary Materials

The following are available online at https://www.mdpi.com/2218-1989/9/10/209/s1, Figure S1. (A) Left panel, PCR with genomic DNA (gDNA) shows presence of intact LIP2p1 or LIP2p2 gene in the WT and absence of such genes in the *lip2p1* (SALK_085456C) and *lip2p2* (SALK_038140C) mutants. Right panel, PCR with gDNA shows presence of the T-DNA in the mutants and its absence in the WT. (B) RT-PCR shows absence of LIP2p1 and LIP2p2 transcripts in both mutants in comparison with control signals from the constitutively expressed 18S ribosomal gene. Figure S2. Comparison of the deduced amino acid sequences and the predicted secondary structures of *Arabidopsis* octanoyltransferases LIP2P1 and LIP2P2. Structure: h, α-helix; s, β-sheet. Residues involved in forming the active site are highlighted in grey and the catalytic lysine and cysteine residues in bold [21]. Table S1. PCR oligonucleotides used in this study.
