# Supplementary material for: Lipidomic Analysis of Plastidial Octanoyltransferase Mutants of Arabidopsis thaliana
Source: Metabolites. 2019 Sep 29;9(10):209. doi: 10.3390/metabo9100209 (PMC6835903; doi:10.3390/metabo9100209)
Supplement: Supplementary file 1 [file metabolites-09-00209-s001.pdf]

Supplementary Material

Table S1. PCR oligonucleotides used in this study.

**Primer**	**Sequence (5´- 3´)**
LBb1.3	ATTTTGCCGATTTCGGAAC
AtLIP2p1-RP	ACTACCAAAGAGGCCCAAAAG
AtLIP2p2-RP	AAGATCCATTTTGTGATTCCG
AtLIP2p1-LP	TGAGGCATTTTAACGAACACC
AtLIP2p2-LP	TCAAAAGGTGGCTCAGAAGTG
AtLIP2p1-transcript-F	GGTCATGGCAGAAGAGCATTGTCG
AtLIP2p1-transcript-R	CACCTTCCTTTTAGCACTCCAATCG
AtLIP2p2-transcript-F	GTTGCAACAAGCTCTGTTACG
AtLIP2p2-transcript-R	TTGTTGCCAACCCAAACTCCA
At18S-F	GGTAGGCGATTGGCTAACATTGTCTGC
At18S-R	GAGACACCAACAGTCTTTCCTCTGCG

Figure S1

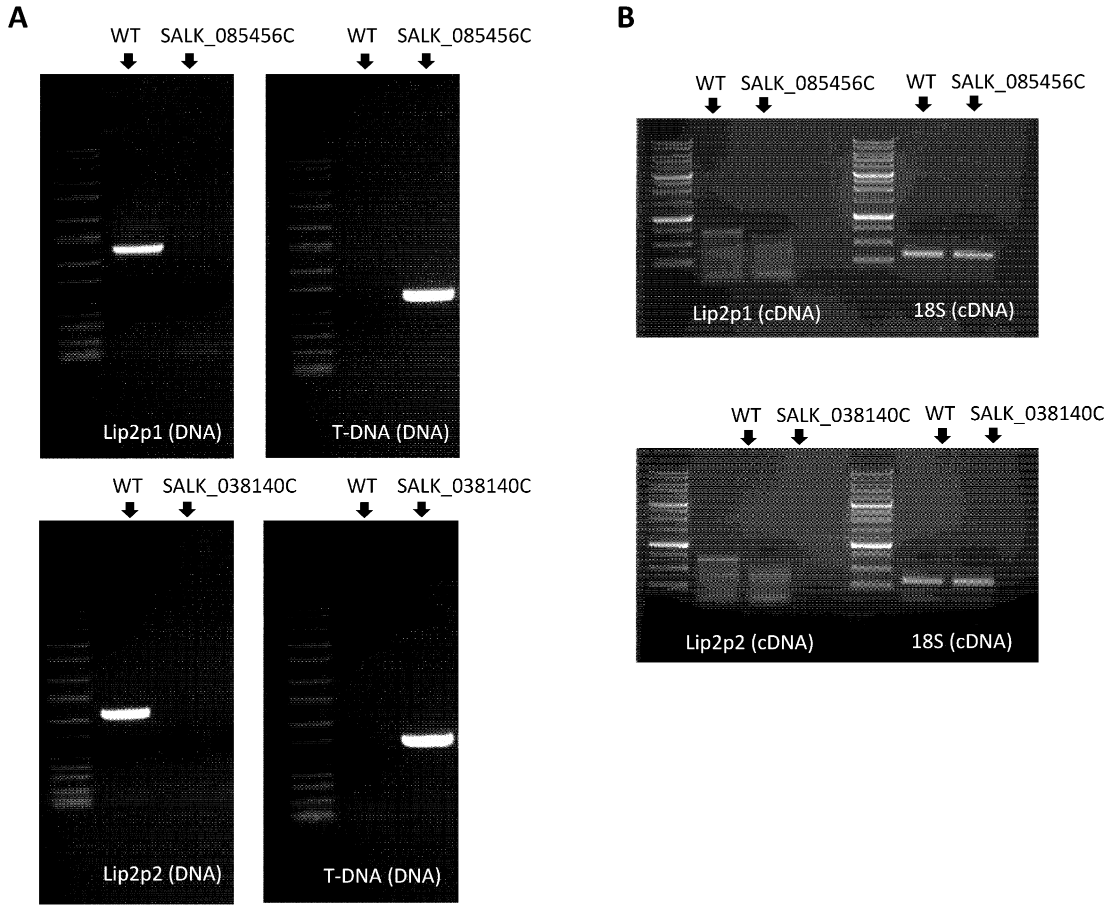

Figure S1. (A) Left panel, PCR with genomic DNA (gDNA) shows presence of intact LIP2p1 or LIP2p2 gene in the WT and absence of such genes in the *lip2p1* (SALK_085456C) and *lip2p2* (SALK_038140C) mutants. Right panel, PCR with gDNA shows presence of the T-DNA in the mutants and its absence in the WT. (B) RT-PCR shows absence of LIP2p1 and LIP2p2 transcripts in both mutants in comparison with control signals from the constitutively expressed 18S ribosomal gene.

Figure S2

```
LIP2P1      MELLNGVETLVSG--IHHHHRTNAKRNRLVRSVKILNSGNHEIPRKCLCFDLYDKLVPYK  58
LIP2P2      MVFSVATSSVTNPKLHHHHHLSDFNRNRVSTSLKIMNSKNHTNPRKCECFDLYDQLIPYK  60
            * :  ...::..    **** :: :***:  *:**:** **  **** ******:*:***
                                                           sss        hh
                                                                      hh

LIP2P1      KAWSWQKSIVEEKKTLIDRNQDCADTVILLQHSPVYTMGTASTEDYLNFDIKDAPFNVYR 118
LIP2P2      KAWSWQKSILNEKKALIDKNQECSDSLIILQHPSVYTMGTGSSENYLNFDIKNAPFDVYR 120
            *********::***:***:**:*:*::*:***..******.*:*:*******:***:***
            hhhhhhhhhh              ssssss    sssss        sss   sss  ss
            hhhhhhhhhh               sssss    sssss                   ss

LIP2P1      TERGGEVTYHGPGQLVMYPIINLRNHEMDLHWYLRMLEEIVIRVLSSTFSIKASRLDGLT 178
LIP2P2      TERGGEVTYHGPGQLVMYPIINLRNHKMDLHWYLRKLEEVVIRVLSSAFAINASRLDGFT 180
            ************************:******** ***:*******:*:*:******:*
            s    sssss  sssssssssss      hhhhhhhhhhhhhhhhh
            s    sssss  sssssssssss      hhhhhhhhhhhhhhhhhh

LIP2P1      GVWVGNQKVAAIGIRVSKWITYHGLALNVTTDLTPFNWIVPCGIRDRKVGNIKGLLEDGE 238
LIP2P2      GVWVGNKKMAAIGIRVSKWMTYHGLALNVTTDLTPFNSIVPCGIRNRGVGSVKGLIEDGE 240
            ******:*:**********:**************** *******:* **.:***:****
             sss  sssssssssss sssssssssss    hhhh                 hhhhh   h
             sss sssssssssssss sssssssssss    hhhh            sss

LIP2P1      HG-MVDDLRLIDIVHESLLKEFSEAFQLQIEKQTVSDPNIL 278
LIP2P2      HYNKLEDLQLLDIAHESLLKEFSEVFQLQMEKQTVFKLEC- 280
            *   ::**:*:**.**********.****:***** . :
            hh-hh     hh   hhhhhhhhhhhh
              hhhhhh   h   hhhhhhhhhhhh
```

Figure S2. Comparison of the deduced amino acid sequences and the predicted secondary structures of Arabidopsis octanoyltransferases LIP2P1 and LIP2P2. Structure: h, α-helix; s, β-sheet. Residues involved in forming the active site are highlighted in grey and the catalytic lysine and cysteine residues in bold [20].
